# Will cases of leprosy reaction increase with COVID-19 infection?

**DOI:** 10.1371/journal.pntd.0008460

**Published:** 2020-07-17

**Authors:** Douglas Eulálio Antunes, Isabela Maria Bernardes Goulart, Luiz Ricardo Goulart

**Affiliations:** 1 National Reference Center for Sanitary Dermatology and Leprosy, Clinics Hospital, Federal University of Uberlandia, Uberlandia, Brazil; 2 Postgraduate Program in Health Sciences, School of Medicine, Federal University of Uberlandia, Uberlandia, Brazil; 3 Institute of Biochemistry and Genetics, Federal University of Uberlandia, Uberlandia, Brazil; 4 Department of Medical Microbiology and Immunology, University of California Davis, Davis, California, United States of America; Faculty of Science, Ain Shams University (ASU), EGYPT

## Introduction

The coronavirus disease 2019 (COVID-19)—caused by severe acute respiratory syndrome coronavirus 2 (SARS-CoV-2), a betacoronavirus (betaCoV)—emerged for the first time as an outbreak of pneumonia in Wuhan, China, and it is now spreading to several countries around the world [1].

The clinical spectrum of COVID-19 ranges from asymptomatic presentation to SARS [2]. The main symptoms of the disease are fever and cough, occurring in 94% and 79% of symptomatic individuals, respectively [3,4]. Elderly individuals are at a higher risk of complications and death associated with COVID-19 because a less vigorous immune response is fatal, especially in those who have comorbidities, such as hypertension, diabetes, coronary heart disease, and chronic obstructive pulmonary disease [4].

The disease has an incubation period that ranges from 5.1 to 11.5 days. The spread of the virus is favored during the asymptomatic period of infected individuals. In addition, SARS-CoV-2 remains viable for 3 hours in the form of aerosols and 72 hours on plastic and stainless steel surfaces [5, 6].

The numbers related to disease incidence are alarming every day, as the total number of deaths increases in proportion to increases in the number of disease cases.

The impact of the pandemic on public health in these countries has been disturbing because the percentage that has advanced to severe forms of the disease will require care in intensive care units. This type of care might cause a collapse in the overall health system.

## Our view

The Brazilian Society of Hansen’s Disease issued an alert on leprosy and COVID-19 coinfection that warned of the risk of infection in those individuals being treated for leprosy reactions. However, further clarification on epidemiological predictions for the COVID-19 infection and its possible role as an immune stimulus triggering leprosy reactions are necessary [7].

Leprosy reactions are immunological events that affect 8% to 33% of leprosy patients, reaching 64.5% in some regions, manifesting before, during, and/or after treatment with multidrug therapy (MDT) [8]. These phenomena are subdivided into type 1 reactions (T1R) and erythema nodosum leprosum (ENL), which is part of the type 2 reaction (T2R). In the T1R, the type 1 helper (Th1) response predominates in lesions and the serum of patients with high levels of cytokines tumor necrosis factor-alpha (TNF-α), interferon-gamma (IFN-γ), interleukin-17 (IL-17), and C-X-C motif chemokine 10 (CXCL10) [9]. Some studies of SARS-CoV-2 infection have reported the presence of a cytokine storm syndrome and a subgroup of patients who progressed to severe forms of the disease, expressing a pro-inflammatory profile in plasma with IL-2, IL-7, TNF-α, and others as significant complications, such as occurs in T1R [10, 11]. Furthermore, the elevation of ferritin and IL-6 were characterized as predictors of fatality for the disease, suggesting that mortality may be caused by hyperinflammation induced by the virus [11]. In relation to ENL, the formation of immune complexes occurs in the blood and deposits in the tissues, especially the skin, kidneys, and joints and is, therefore, a type III hypersensitivity reaction [12]. An extensive neutrophil infiltration in pulmonary capillaries may be induced by COVID-19, evidenced in an autopsy specimen from lungs of severe patients [13]. Neutrophils are associated with ENL, since skin lesions present an intense perivascular infiltrate of neutrophils throughout the dermis, and these cells are able to trigger ENL, allowing TNF-α and IL-8 release after stimulation with lipopolysaccharide (LPS) [14,15]. We support that neutrophils, influenced by COVID-19, may propitiate ENL in those infected patients.

In both reactions, we warn of the possible effect that COVID-19 infection may have on the number of cases of these immunological events because the presence of infection is an important risk factor for triggering leprosy reactions [8].

Another disturbing factor, which may contribute to the susceptibility of those affected by leprosy reactions, are the treatments implemented during these events that interfere with the inflammatory response of these patients.

In the treatment of T1R, prednisone is used at a dose of 1 mg/kg and sometimes even higher (1.5 mg/kg) [16]. Prednisone is a drug similar to the endogenous hormone cortisol that allows several effects, such as anti-inflammatory, immunosuppressive, anti-proliferative, and vasoconstrictive actions [17]. The genetic mechanism of this drug is related to interference in the transcription of nuclear factor kappa B (NF-κB), which may cause suppression of the synthesis of pro-inflammatory cytokines, such as IL-1, IL-2, IL-6, IL-8, TNF-α, IFN-γ, and vascular endothelial growth factor (VEGF) [17]. The anti-inflammatory and immunosuppressive effects of glucocorticoids are dose dependent. Therefore, at high doses, their effects are immunosuppressive, causing emerging infections, such as COVID-19, in these susceptible patients [17].

In ENL, thalidomide is prescribed, an immunomodulatory drug that inhibits the expression of TNF-α and IFN-γ, affecting the pro-inflammatory activity of these patients. It also interferes in response to other microorganisms, such as COVID-19, which also may favor the manifestation of severe forms referring to the clinical spectrum of this disease [18]. Despite the fact that some T2R patients may express pro-inflammatory cytokines in several tissues, the expression of IL-10 may be elevated in those individuals without the use of thalidomide, indicating *Mycobacterium leprae* viability, which can contribute to a low immune response to COVID-19 in these individuals [19].

Even though some clinical trials are underway to investigate the use of thalidomide—alone or combined with systemic glucocorticoids, such as methylprednisolone—to treat moderate and severe COVID-19 pneumonia, there are no final conclusions ensuring that they are an effective treatment [20].

Thus, we believe that, as the number of new cases of COVID-19 infection increases, the incidence of leprosy reactions may also increase considerably. In addition, it is worth remembering that reactive patients are treated with drugs that affect the stability of the immune system, which, in turn, can contribute to the manifestation of SARS, especially in those who are elderly with comorbidities. It is important to remember that, as early as possible, social isolation measures should be started to flatten the curve and reduce the number of new COVID-19 cases, consequently preventing an unprecedented collapse of all levels of care and guaranteeing the monitoring of leprosy reactions.

Finally, for leprosy patients on treatment or after discharge from MDT, we recommend what medical doctors and health teams advise with the goal to avoid exposure to SARS-CoV-2, such as the following: the use of masks in the community; stay home and self-isolate from others; wash hands regularly for 20 seconds, with soap and water or alcohol-based hand rub; avoid close contact (staying 2 meters away from other people); and cover your nose and mouth with a disposable tissue or flexed elbow when you cough or sneeze. These precautions will aid prevention of the spread of COVID-19 infection—and its severe manifestations, including leprosy reactions.
